# Different effects of prenatal MAM vs. perinatal THC exposure on regional cerebral blood perfusion detected by Arterial Spin Labelling MRI in rats

**DOI:** 10.1038/s41598-019-42532-z

**Published:** 2019-04-15

**Authors:** Eva Drazanova, Jana Ruda-Kucerova, Lucie Kratka, Tibor Stark, Martin Kuchar, Michal Maryska, Filippo Drago, Zenon Starcuk, Vincenzo Micale

**Affiliations:** 1 Department of Pharmacology, Faculty of Medicine, Masaryk University, Brno, Czech Republic; 2 Institute of Scientific Instruments of the Czech Academy of Sciences, Brno, Czech Republic; 3 Department of Biomedical Engineering, Faculty of Electrical Engineering and Communication, University of Technology, Brno, Czech Republic; 4 Forensic Laboratory of Biologically Active Substances, Department of Chemistry of Natural Compounds, University of Chemistry and Technology Prague, Prague, Czech Republic; 5 Department of Biomedical and Biotechnological Sciences, Section of Pharmacology, School of Medicine, University of Catania, Catania, Italy; 6 National Institute of Mental Health, Klecany, Czech Republic

## Abstract

Clinical studies consistently report structural impairments (i.e.: ventricular enlargement, decreased volume of anterior cingulate cortex or hippocampus) and functional abnormalities including changes in regional cerebral blood flow in individuals suffering from schizophrenia, which can be evaluated by magnetic resonance imaging (MRI) techniques. The aim of this study was to assess cerebral blood perfusion in several schizophrenia-related brain regions using Arterial Spin Labelling MRI (ASL MRI, 9.4 T Bruker BioSpec 94/30USR scanner) in rats. In this study, prenatal exposure to methylazoxymethanol acetate (MAM, 22 mg/kg) at gestational day (GD) 17 and the perinatal treatment with Δ-9-tetrahydrocannabinol (THC, 5 mg/kg) from GD15 to postnatal day 9 elicited behavioral deficits consistent with schizophrenia-like phenotype, which is in agreement with the neurodevelopmental hypothesis of schizophrenia. In MAM exposed rats a significant enlargement of lateral ventricles and perfusion changes (i.e.: increased blood perfusion in the circle of Willis and sensorimotor cortex and decreased perfusion in hippocampus) were detected. On the other hand, the THC perinatally exposed rats did not show differences in the cerebral blood perfusion in any region of interest. These results suggest that although both pre/perinatal insults showed some of the schizophrenia-like deficits, these are not strictly related to distinct hemodynamic features.

## Introduction

Schizophrenia (SCZ) is a debilitating mental illness condition affecting both brain structure and function^1^. A meta-analysis of clinical studies confirmed consistently reported progressive ventricular (lateral and third) enlargement, along with decreased volume of amygdala, anterior cingulate cortex, frontal and temporal lobes, hippocampus and thalamus^2^. These changes are paralleled by functional abnormalities detected by neuroimaging methods. A meta-analysis of functional neuroimaging studies employing nuclear medicine imaging techniques (PET, SPECT) or blood-oxygen level determination MRI reported reduced activation in the insula/superior temporal gyrus and the medial frontal/anterior cingulate cortex in SCZ suffering patients. Noteworthy, these changes were partly worsened by antipsychotic treatment^1^. The ASL MRI approach represents a quantitative method for measuring actual cerebral blood flow (CBF), which could be used as a marker for the functional state of the brain tissue in both human and animal studies^3,4^. However, previous ASL studies in SCZ suffering individuals are characterized by contradictory findings (i.e.: increased, decreased or no difference in the CBF) based on different stages of the disease, on the selection of brain regions of interest (ROIs), severity of symptoms, pharmacological treatment, and last but not least on patients recruitment^5–7^. Conversely, the standardized conditions in preclinical investigation, despite its limits *per se*, allow to assess both the development of SCZ-like symptoms and the potential effect of antipsychotic treatment independently on CBF, which is not easy achievable in clinical research.

Currently, the neurodevelopmental hypothesis of SCZ is widely accepted^8,9^ and several animal models based on prenatal environmental insults [i.e.: polyinosinic:polycytidylic acid: poly(I:C), methylazoxymethanol (MAM) acetate] have been developed and well validated^10,11^. Furthermore, increasing evidence from both preclinical and human studies suggests that the heavy use of THC during pregnancy could increase the risk of developing neuropsychiatric disorders at adulthood, including SCZ^12–16^. However, human studies, in particular those using retrospective evaluations, have some limitations because of the vast heterogeneity of cannabis intake^17^. Hence, research with laboratory animals enables the study of specific effects of cannabinoids during early stages of development. Importantly, the pre/perinatal period may be a stage of particular vulnerability, which allows the assessment of long lasting effects of THC in the development of neuropsychiatric-like disorders in adulthood^12–14^.

There is an almost complete lack of functional neuroimaging studies in animal models of SCZ (or animal model of psychopathology), therefore, the aim of this study was to assess potentially altered CBF in several SCZ-related brain regions using (1) a validated neurodevelopmental model of SCZ induced by the prenatal MAM exposure and (2) the perinatal treatment with THC potentially leading to a SCZ-like phenotype. We have selected the ROIs according to our previous study where we have observed a significant enhancement of perfusion in the circle of Willis, the hippocampus and the sensorimotor cortex in the poly(I:C) neurodevelopmental model of SCZ^18^. The circle of Willis was included as a region that reflects blood perfusion in the whole brain^18,19^. We have further assessed two other brain regions highly relevant for SCZ – the anterior cingulate cortex which is part of the prefrontal cortex^1,20,21^ and the striatum, i.e. the caudate putamen^2,22,23^.

## Material and Methods

### Animals

Timely mated female albino Sprague-Dawley rats were purchased from Charles River (Germany) at gestational day (GD) 13 and housed individually. They were randomly assigned to the MAM or the THC experimental group. Environmental conditions during the whole study were constant: relative humidity 50–60%, temperature 23 °C ± 1 °C, normal 12-hour light-dark cycle (light on 7 a.m. to 7 p.m.). Food and water were available ad libitum. All procedures were performed in accordance with EU Directive No. 2010/63/EU and approved by the Animal Care Committee of the Faculty of Medicine, Masaryk University, Czech Republic and the Czech Governmental Animal Care Committee, in compliance with Czech Animal Protection Act No. 246/1992.

### Drugs

Methylazoxymethanol acetate (MAM; Midwest Research Institute, Kansas City, USA) was dissolved in saline and administered intraperitoneally at a dose of 22 mg/kg in 1 ml/kg volume on GD 17. Saline was injected to the control (CTR) group as vehicle.

Δ-9-tetrahydrocannabinol (THC, 10 mg/ml in ethanol solution) was obtained from University of Chemistry and Technology, Prague^24^. THC was prepared according to the published procedure from cannabidiol (4.00 g, 12.7 mmol) as light brown thick oil (2.04 g, yield 51%). The purity of THC (as determined by HPLC) was 97.2%. The starting material cannabidiol (purity 99.7%) has a natural origin and was isolated from the plant *Cannabis Sativa* L.^25^. For oral administration THC was dissolved in sesame oil (5 mg/kg in 1 ml) and prepared as previously described^26^. Sesame oil was administered to the CTR group as vehicle.

### Experimental models

#### Prenatal MAM exposure model

The MAM model was induced as previously described^27–32^. Briefly, MAM (22 mg/kg; i.p.) or vehicle (CTR: saline) were administered intraperitoneally on GD 17. No cross-fostering was used, the mothers were regularly weighted and no differences in the body weight gains were observed between CTR and MAM treated dams. Furthermore, prenatal MAM exposure did not affect pregnancy length, litter size at birth, pup weight gain, and postnatal mortality (data not shown). The offspring were weaned on postnatal day (PND) 22 and housed in groups of 2–3. The behavioral tests and MRI scanning were performed in male offspring at adulthood (4 months of age). There were 8 CTR rats from 5 different litters and 6 MAM-exposed rats from 4 litters.

#### Perinatal THC exposure model

The perinatal THC exposure was performed as previously described^33,34^. Pregnant rats received a daily dose of 5 mg/kg THC or vehicle (CTR: sesame oil) administered through an oral gavage from GD 15 to PND 9. The administered dose is equivalent to the current estimates of moderate exposure to THC in humans, correcting for differences in the route of administration and the body surface area^26^. No cross-fostering was used, the mothers were regularly weighed and no differences in the body weight gains were observed between the CTR and the THC treated dams. Furthermore, the perinatal exposure did not affect pregnancy length, litter size at birth, pup weight gain, and postnatal mortality (data not shown). The offspring were weaned on PND 22 and housed in groups of 2–3. The behavioral tests and MRI scanning were performed in male offspring at adulthood (6 months of age). There were 10 CTR rats from 3 different litters and 10 THC-exposed rats from 4 litters.

### Behavioral testing

#### Social interaction test (SIT)

The test was carried out in a moderately illuminated room, as previously described^32,35^. Each animal was allowed to freely explore an unfamiliar congener in a metal arena (60 × 60 × 60 cm) for 10 min. The arena was cleaned with 0.1% acetic acid and dried after each trial. Social behaviors were defined as sniffing, following, grooming, mounting, and nosing. The whole testing phase was recorded and analyzed by two observers blind to the treatment groups who scored the time spent in social behaviors and the number of interactions.

#### Novel object recognition test (NORT)

The experimental apparatus used for the NOR test was an arena (45 × 45 × 30 cm) made of Plexiglas, placed in a moderately illuminated room. As previously described^32,35^, each animal was placed in the arena and allowed to explore two identical previously unseen objects for 5 min (familiarization phase). After an inter-trial interval of 3 min, one of the two familiar objects was replaced by a novel, previously unseen object and rats were returned to the arena for the 5 min test phase. During the test phase, the time spent exploring the familiar object (T_f_) and the new object (T_n_) was videotaped and analyzed separately by two observers blind to the treatment groups, and the discrimination index was calculated as follows: discrimination index $=\frac{({T}_{n}-{T}_{f})}{({T}_{n}+{T}_{f})}$. The arena and all objects were cleaned with 0.1% acetic acid and dried after each trial.

### Magnetic resonance imaging

**Figure 1:**
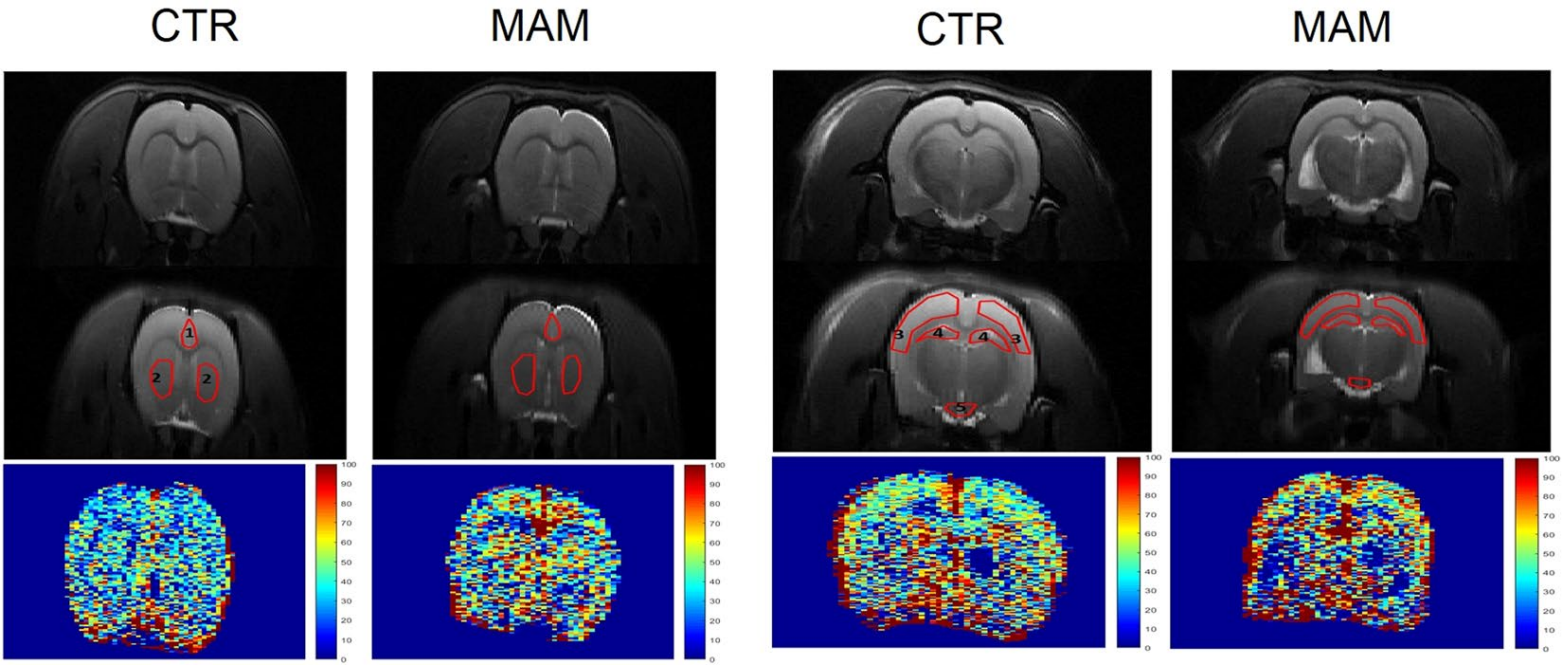
ASL image in the MAM model. The figure shows anatomical (T2 weighted) images in the top part, the delineation of regions of interest for the ASL analysis (central part) and perfusion map (bottom part) in one representative animal from each CTR and MAM exposed group. ROIs are numbered as follows. At +1.6 mm position from bregma: 1 – prefrontal cortex, 2 – caudate putamen; at −3.14 mm position from bregma: 3 – sensorimotor cortex, 4 – hippocampus, 5 – circle of Willis.

**Figure 2:**
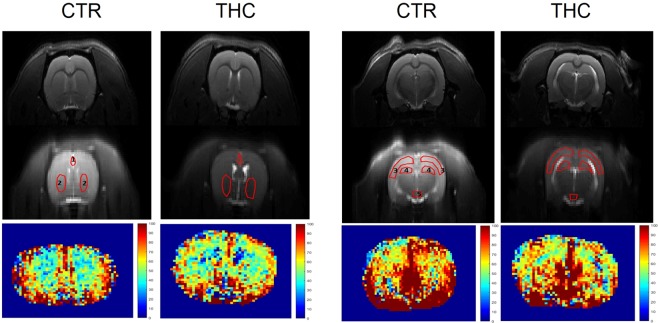
ASL image in the THC model. The figure shows anatomical (T2 weighted) images in the top part, the delineation of regions of interest for the ASL analysis (central part) and perfusion map (bottom part) in one representative animal from each CTR and THC exposed group. ROIs are numbered as follows. At +1.6 mm position from bregma: 1 – prefrontal cortex, 2 – caudate putamen; at −3.14 mm position from bregma: 3 – sensorimotor cortex, 4 – hippocampus, 5 – circle of Willis.

**Figure 3:**
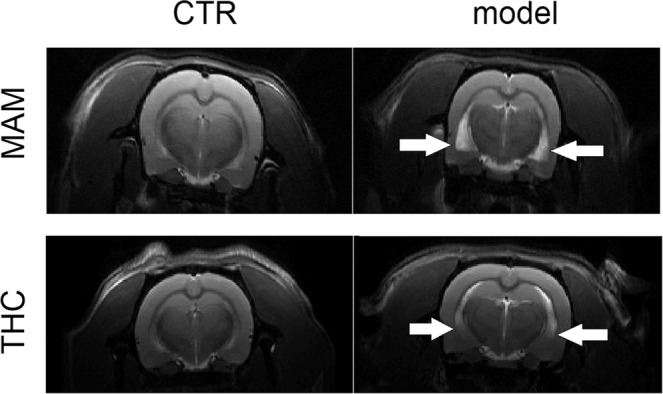
Lateral ventricles in the MAM and THC models. The figure shows anatomical (T2 weighted) images with visible ventricle enlargement present in MAM and THC exposed animals and their respective controls (CTR). White arrows point to the edges of the enlarged lateral ventricles.

All animals were scanned once. The ROIs were selected based on high relevance for SCZ^1,18,19,21–23,36,37^ (Figs 1 and 2), together with the need of assessing two slices of the brain. The first slice was positioned at +1.6 mm from bregma and the second selected slice was positioned at −3.14 mm from bregma^38^. The anatomical slice positioned at −3.14 mm from bregma was also used for lateral ventricles (Fig. 3) quantification. The selection of two slices allowed for short time of MRI examination and a relatively short general anesthesia (approximately 40 minutes) which is unlikely to induce any strong brain perfusion aberrations^39^.

MRI was performed on a 9.4 T Bruker BioSpec 94/30USR scanner with 2 × 2 surface array rat head received coil and a volume transmitter coil. The measurement was conducted under 2% isoflurane anesthesia and 1000 ml/min of oxygen. The animals were laid on a thermal pad and their body temperature and respiratory curve were monitored during the measurement process. T2-weighted anatomical images were taken using the RARE sequence with TR = 3500 ms, TE = 36 ms, FOV 40.3 mm × 30.5 mm, image matrix 256 × 256. Fifteen axial slices with thickness of 1.25 mm were acquired; and the slices covered the brain from the root of the olfactory bulbs to the cerebellum. These anatomical images provided information for the lateral ventricles area evaluation and the background for the selection of an axial slice suitable for the ASL sequence, for which the prefrontal cortex, sensorimotor cortex, piriform cortex, hippocampus, caudate putamen and the circle of Willis were the ROIs (depicted in Figs 1 and 2). In these measurements, one axial slice with a thickness of 1.25 mm was imaged with a FAIR-RARE, which sequence was applied with TR = 10000 ms, TE = 37.78 ms, TI stepped through 30, 50, 100, 200, 300, 500, 700, 900, 1000, 1100, 1500, 1800, 2200, 2800, 3200 ms, FOV 40.3 mm × 30.5 mm, image matrix 128 × 96, adiabatic inversion pulse length 16 ms, bandwidth 4866.2 Hz, inversion slab thickness 5 mm. By repeating the measurement twice, with slice-selective and nonselective inversion, two images were obtained, from which the perfusion map was calculated according to the following Equation^4^.

$${\rm{CBF}}={\rm{\lambda }}\cdot \frac{{{\rm{T}}}_{1,{\rm{nonsel}}}}{{{\rm{T}}}_{1,{\rm{blood}}}}(\frac{1}{{{\rm{T}}}_{1,{\rm{sel}}}}-\frac{1}{{{\rm{T}}}_{1,{\rm{nonsel}}}}),$$

where CBF is the CBF (usually expressed in mL/min in 100 g of tissue), λ is the blood-brain partition coefficient, expressing the ratio of the quantity of water per gram of tissue to the quantity of water per milliliter of blood, which is known to be 0.89 ± 0.03 mL(blood)/g(tissue) in the rat brain^40^. T_1,nonsel_ and T_1,sel_ are the apparent longitudinal relaxation times derived from the image series applying nonselective and slice-selective inversions, respectively, T_1,blood_ is the longitudinal relaxation of capillary blood.

#### MRI data analysis

The lateral ventricles quantification was evaluated in Marevisi 8.0 (Institute for Biodiagnostics, National Research Council, Canada). ROIs were manually outlined on anatomical image^41^ and lateral ventricular areas were calculated for each animal using pixel counts converting into actual area. Figure 3 shows the representative images of lateral ventricles, which were robustly enlarged in the MAM model while in the THC exposed rats just a subtle lateral ventricles enlargement was observed.

The perfusion maps were calculated in Paravision 5.1 (Bruker Biospin, Ettlingen, Germany) and further analyzed in manually drawn brain ROI by a blinded researcher using our own MATLAB R2010a code (The MathWorks Inc., Natick, MA, USA) according to the rat brain atlas^38^. Figure 1 shows the anatomical images, delineation of the ROIs for the ASL analysis and perfusion maps of one representative MAM and CTR animal. Figure 2 analogically demonstrates the MRI images of the THC model.

All ROIs except for the prefrontal cortex and the circle of Willis were evaluated as the average of the left and right hemisphere value.

### Statistical data analysis

Primary data were summarized using arithmetic mean and standard error of mean (±SEM). The behavioral and MRI variables were compared by a t-test or Mann-Whitney U (MWU) test depending on the result of Kolmogorov-Smirnov test of normality. Furthermore, relevant correlations between the body weight and the perfusion of selected brain regions were calculated using Pearson correlation for parametric data and Spearman rank for non-parametric data. All analyses were calculated using Statistica 13.2 (StatSoft, USA), value p < 0.05 was recognized as the boundary of statistical significance in all applied tests.

## Results

### Prenatal MAM exposure model

Behavioral phenotype was assessed by SIT in order to evaluate potential social deficits and NORT to establish short term recognition memory as representative measure of negative- and cognitive-like symptoms of SCZ. MAM prenatally exposed rats showed social deficit in the SIT in terms of time spent by social behaviors (t-test, *p* = 0.005). The number of interactions with the partner was equal as in the CTR group (t-test, not significant). As expected, MAM rats also exhibited a cognitive deficit in the NORT measured by decreased discrimination index (MWU test, *p* = 0.022).

Neuroimaging anatomical data showed a significant enlargement of the lateral ventricles. The mean value of the MAM exposed animals lateral ventricles area was 0.13 ± 0.01 mm^2^ and 0.03 ± 0.05 mm^2^ in the CTR rats. MWU test indicated significantly enlarged lateral ventricles area in the MAM prenatally exposed rats (*p* = 0.010).

**Figure 4:**
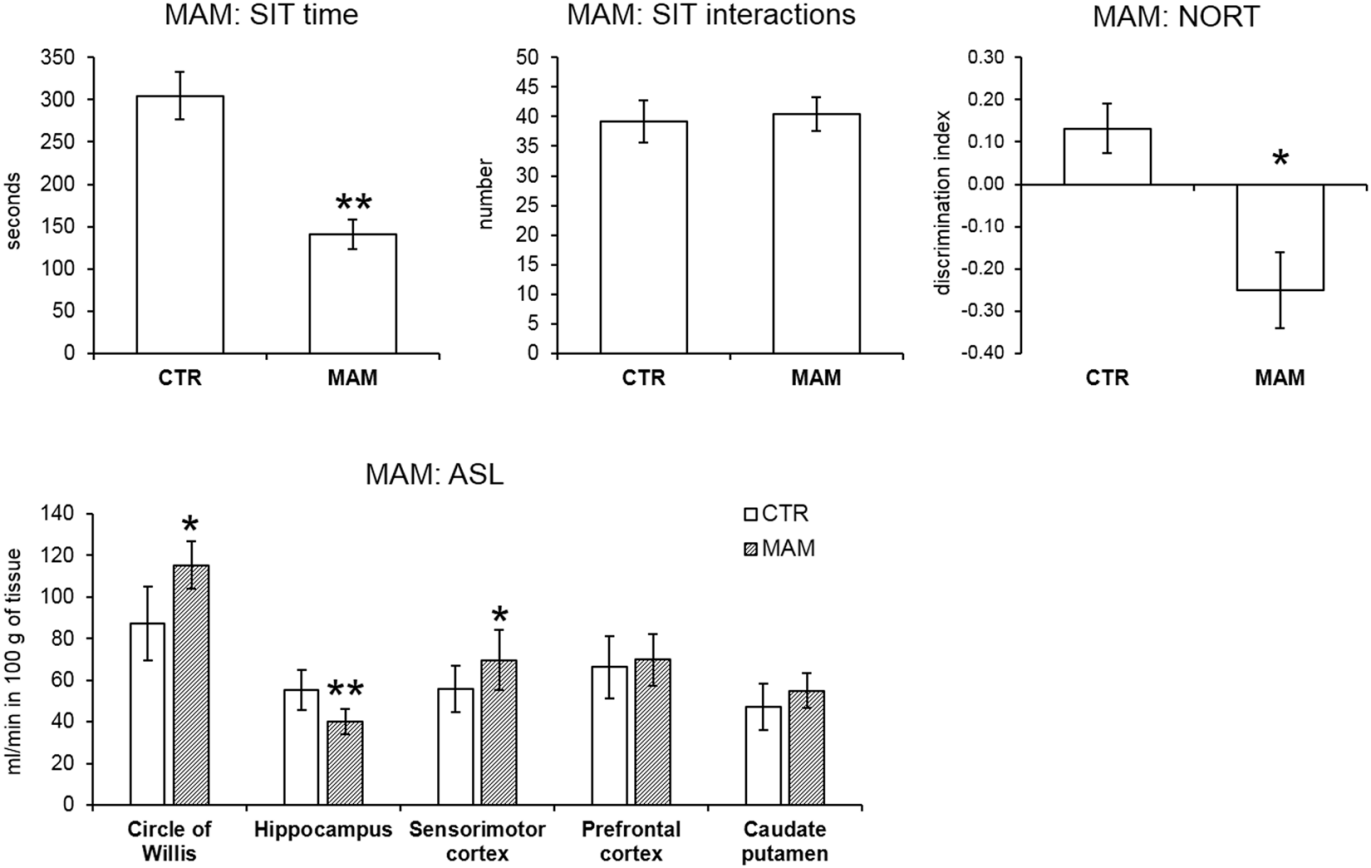
Behavior and cerebral blood perfusion in the MAM model. The bar graphs indicate the mean ± SEM of all variables. MAM rats spent shorter time by social interactions in the SIT but the number of interactions did not differ. The short-term recognition memory deficit in the MAM rats detected by NORT is apparent by a decreased discrimination index. In the analysis of regional blood perfusion MWU test revealed significantly higher perfusion of circle of Willis, and sensorimotor cortex in the MAM animals while hippocampus perfusion was lower (*p ≤ 0.05, **p ≤ 0.01).

Neuroimaging functional data showed significant differences in all ROIs. MWU test indicated significantly higher perfusion in the circle of Willis (*p* = 0.020) and the sensorimotor cortex (*p* = 0.043) while the perfusion of the hippocampus was decreased (*p* = 0.003). No difference was found in the perfusion of the prefrontal cortex (t-test, not significant) and the caudate putamen (t-test, not significant). Furthermore, Spearman rank did not identify any significant correlations between the circle of Willis and any other ROIs in neither the CTR nor the MAM prenatally exposed rats, which could be due to the small sample size (n = 6). Behavioral and ASL data are presented in Fig. 4.

### Perinatal THC exposure model

THC exposed rats exhibited analogous behavioral phenotype as the MAM rats: they spent less time by social behaviors in the SIT (MWU test, *p* = 0.009) while the number of interactions with the partner did not differ (t-test, not significant). THC rats also showed a cognitive deficit in the NORT measured by decreased discrimination index (MWU test, *p* = 0.009).

Neuroimaging anatomical data did not show significant lateral ventricles enlargement in the THC model. The mean value of the THC exposed animals’ lateral ventricles area was 0.02 ± 0.03 mm^2^ and 0.01 ± 0.02 mm^2^ in the CTR animals. MWU test did not indicate any significantly enlarged lateral ventricles area in the THC rats. Even though lateral ventricles seemed to be subtle enlarged visually (Fig. 3).

**Figure 5:**
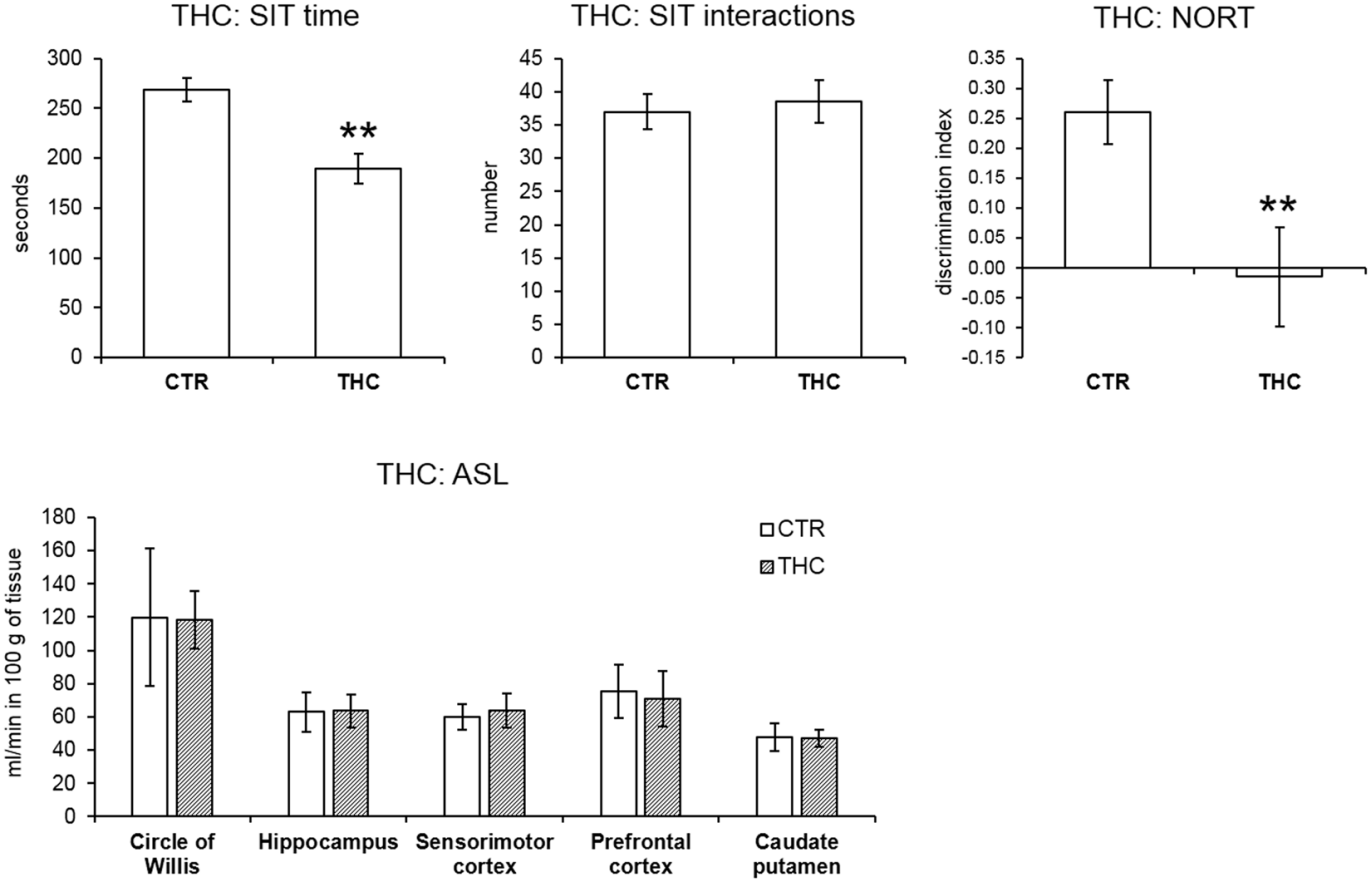
Behavior and cerebral blood perfusion in the THC model. The bar graphs indicate the mean ± SEM of all variables. THC rats spent shorter time by social interactions in the SIT but the number of interactions did not differ. The short-term recognition memory deficit in the THC model detected by NORT is apparent by a decreased discrimination index. In the analysis of regional blood perfusion t-test did not indicate any significant differences between the groups.

Neuroimaging functional data did not show significant differences in any of the ROIs. Pearson correlation in CTR rats indicated a significant association between the perfusion of the circle of Willis and those of the hippocampus (*p* = 0.014, r = 0.743), and the caudate putamen (*p* = 0.029, r = 0.685) and a trend to significance in analysis of the circle of Willis with sensorimotor cortex (*p* = 0.071, r = 0.593). On the other hand, in the THC exposed animals only the association between the perfusion of the circle of Willis and hippocampus was present (*p* = 0.009, r = 0.773) while the correlations between the perfusion of circle of Willis and the sensorimotor cortex (*p* = 0.125, r = 0.518) and caudate putamen were absent (*p* = 0.078, r = 0.581). This may indicate certain hemodynamic dysregulation in the cortical regions. Behavioral and ASL data are presented in Fig. 5.

### Age related changes in CTR groups

**Figure 6:**
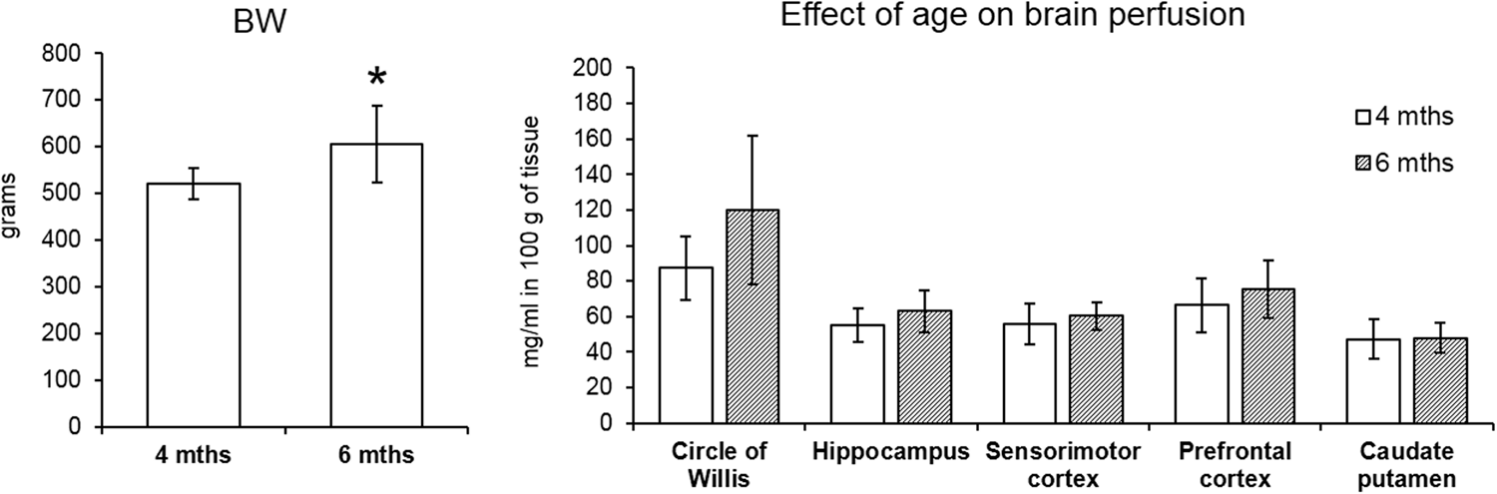
Evaluation of potential age related effects in control rats. The bar graphs indicate the mean ± SEM of all variables. T-test revealed significantly higher body weight in the older animals but cerebral perfusion did not differ between the groups in any ROIs (*p ≤ 0.05).

In order to establish consistency of the ASL data, CTR groups of both the MAM and the THC exposure models were compared. The rats in the MAM experiment were scanned at the age of 4 months while the animals in the THC model were older – 6 months. The age difference was reflected in the body weight, the older rats weighed significantly more, as proven by a t-test, *p* = 0.018 (Fig. 6). However, brain perfusion did not differ between the CTR groups in any ROIs and these values did not correlate with body weight (Pearson correlation).

## Discussion

In this study, we observed consistent behavioral changes in two different experimental models of perinatal insult, which could resemble a SCZ-like phenotype. Rat males in both models (MAM and THC) showed social withdrawal in social interaction and cognitive impairment in a short-term recognition task, which are often considered the two signs of SCZ- like symptoms^42^. Our behavioral data further confirmed the effects of prenatal MAM exposure on the development of SCZ-like phenotype in agreement with the neurodevelopmental hypothesis of SCZ^8,43^. Interestingly, the MAM induced behavioral deficits were paralleled by substantial dysregulation of regional CBF. The THC experimental model was based on the hypothesis that perinatal exposure to cannabis may impact the normal developmental trajectories in the CNS, thereby resulting in neuropsychiatric disorders including SCZ^15^. Although our behavioral data confirmed that THC exposed rats showed a phenotype similar to the MAM model; nevertheless, the behavioural assays (i.e., SIT and NORT) used in the present study are not strictly specific for SCZ and could be applicable to assess symptoms domains shared with other neuropsychiatric disorders (i.e. autism, depression or anxiety)^32^. Thus, we could conclude that pre/perinatal MAM or THC exposure led to behavioural impairments which are consistent with neuropsychiatric disorders, including SCZ^32^. Further behavioural tasks to assess different cognitive or social-like deficits would be useful. However, the regional CBF pattern was showed no apparent changes in the THC model. Furthermore, we have observed a robust enlargement of lateral ventricles in the MAM rats while the THC exposed rats showed a non-significant only a subtle structural impairment in the lateral ventricle region. These results are discussed in context of other studies in the following sections.

### Regional CBF changes in the MAM and THC model

In the MAM prenatally exposed rats, we revealed significant perfusion changes showing a higher CBF in the circle of Willis and the sensorimotor cortex together with decreased perfusion of the hippocampus. On the other hand, the THC perinatally exposed rats did not show differences in the CBF in any ROIs. Importantly, we scanned both rat models at adulthood but there was an age difference between them, i.e. MAM and their CTR rats were 4 months old while THC animals 6 months old. In order to CTR for potential age-related effects, we compared the regional CBF and body weight between the CTR groups from both experimental models. In this analysis, we have identified a significant difference in the body weight but not in the CBF. Furthermore, body weight did not correlate with CBF in any brain region. This indicates that cerebral perfusion is unlikely to be associated with body weight and it seems to be consistent at this age difference. Similarly, in our previous study we have detected an increased perfusion in male rats compared to females in all analyzed brain regions^18^ but the correlation between brain perfusion and body weight was not present either (unpublished findings).

The field of functional neuroimaging in animal models of psychopathology included SCZ remains understudied. To the best of our knowledge, this is the first study focusing on ASL in the MAM and THC models. We have identified only two relevant papers for comparison: our own previous study using the poly(I:C) neurodevelopmental model^18^ and an experiment on phencyclidine model and neonatal ventral hippocampal lesion (NVHL) model^44^. The poly(I:C) prenatally challenged animals showed increased perfusion in the circle of Willis as the region which represents the main blood supply for the brain^19^, hippocampus, and sensorimotor cortex^18^. On the other hand, higher perfusion of the entorhinal-piriform cortex, the shell part of the nucleus accumbens and the ventral pallidum and lower CBF in the temporal cortex and partly the prefrontal cortex were reported in both the phencyclidine and the NVHL models^44^.

These results are difficult to compare because different ROIs were selected, but CBF dysregulation seems to be a common feature. In this study, we observed increased perfusion of the sensorimotor cortex in the adult MAM offspring. Importantly, CBF changes in the MAM and poly(I:C) models show increased perfusion of the cortical areas which seems to be a common feature of the these SCZ-like phenotypes^18^. Interestingly, prenatal MAM exposure was found to possess certain anti-angiogenic effects leading to lower density of cortical capillaries at adulthood which may contribute to CBF changes^45^. However, the exact selection of cortical region seems to be crucial as in this study no perfusion change was present in the part of the prefrontal cortex.

#### Absence of regional CBF changes in THC model

Despite the presence of consistent behavioral phenotype, we detected no significant perfusion changes in the THC model. The only sign of potential hemodynamic alteration in the THC exposed rats was the lack of correlation between the CBF of the circle of Willis and of the sensorimotor cortex/caudate putamen. Thus, we could speculate that it may be a sign of CBF dysregulation as previously suggested earlier in the study using poly(I:C) neurodevelopmental model^18^. Out of all animal models of SCZ where CBF was studied, the THC model seems to be the least affected, as judged by the functional perfusion and structural changes. This model is based on the hypothesis that perinatal exposure to THC could induce psychiatric disorders later in life^46^, also by modulating several neurotransmitters system (DAergic, Glutamatergic and/or GABAergic) via central cannabinoid type 1 receptor^47^. Despite the positive behavioral outcome, our CBF data indicate that the THC model is hemodynamically stable, which is an unexpected result based on findings from other animal models^18,44^. This may limit its validity for neuroimaging studies of SCZ. Importantly, this study has included a number of ROIs, but we cannot rule out the possibility of CBF changes in other brain regions.

The data from the THC perinatally treated rats might be partly interpreted in a context of studies employing acute or chronic THC (or cannabis) dosing at adulthood. In rats, an autoradiographic study evaluated dose-dependent effect of THC on CBF in a large number of brain regions^48^. The brain areas with deficient CBF were the CA1 region of hippocampus, frontal and medial prefrontal cortex, nucleus accumbens, and claustrum while regions where no change was observed were medial septum, ventral tegmental area, caudate, temporal, parietal and occipital cortex, and cerebellum^48^. These data are in agreement with our THC model in the caudate and the sensorimotor cortex, but they differ in the prefrontal areas and the hippocampus. In the case of hippocampus, the difference could be ascribed to the definition of the region and its subdivision in the radiographic study.

Importantly, the finding of no excessive CBF alterations in the autoradiographic study^48^ is not in line with clinical studies of CBF after an acute dose of THC, consistently reporting CBF increase in the prefrontal regions and the basal ganglia and a CBF decrease in the frontal, parietal, temporal, and occipital lobes in chronic cannabis users^49^. Taken together, the preclinical findings of hemodynamic changes induced by THC are inconsistent and biased by different species and phytocannabinoids used^50^.

A clinical PET study employing acute doses of THC in healthy volunteers showed transiently increased cerebral blood perfusion in the frontal, insular and anterior cingulate regions^51^. Another clinical trial with abstaining chronic cannabis users indicated higher CBF in the right pallidum/putamen of the cannabis users compared with nonusers. In this study, regional cerebral blood perfusion of right the superior frontal cortex correlated positively with THC levels in urine^52^. These findings indicate that THC is indeed able to alter CBF. However, these effects are likely to be dependent on dose and repeated exposure. Therefore, perinatal THC treatment may not lead to alterations of CBF persisting to adulthood as observed in our study.

#### Comparison of the results to clinical evidence

In clinical studies similar perfusion dysregulations as in the MAM or poly(I:C) model^18^ were observed using several imaging techniques. ASL MRI detected hyperperfusion in the cerebellum, brainstem, and thalamus and hypoperfusion in the frontal lobes bilaterally, in the anterior and medial cingulate gyri, and in the parietal lobes bilaterally in patients suffering SCZ^53^. A similar pattern of temporal and frontal hypoperfusion in SCZ affected individuals was registered in the SPECT studies^54,55^. Furthermore, there is also a PET study connecting positive and negative psychiatric symptoms with specific brain regions. Positive symptoms were associated with increased blood perfusion in the anterior cingulate cortex, the cingulate and the superior frontal gyrus and decreased perfusion of the hippocampus, the precentral and middle frontal gyrus. Negative symptoms were linked to the increased perfusion of the frontal and the parietal cortex, the cingulate and the left middle frontal gyrus^56^. However, such data are difficult to put in the context of preclinical animal models. The inconsistences are caused mainly by the lack of studies using first-episode SCZ affected or drug-naive patients and variables resulted from the different ROIs selection and/or different imaging techniques^57^.

### Enlargement of lateral ventricles

In our study, robust ventricular enlargement as one of the progressive features seen in SCZ affected patients^58^ was present in MAM exposed animals while just subtle ventricular enlargement was detected visually in case of THC animals. Consistently with clinical evidence, ventricle enlargement is a commonly observed feature in animal models of SCZ such as the MAM model^41^, poly(I:C)^18^, NVHL model^59^ in rats or even some genetic mouse models^60^. So far, we can just speculate whether hemodynamic changes could precede structural impairment^55^ and significant CBF dysregulation could play a role in the progression of ventricular enlargement. The almost complete lack of functional and structural abnormalities in the behaviorally valid THC model observed in this study may indicate a different validity profile of this model in context of SCZ research. Still, there is a big heterogeneity of brain structure anomalies in human patients suffering SCZ as well^61,62^, hence the THC model may be useful in other studies not focusing on brain function or structure.

## Conclusion and Future Perspective

Although pre/perinatal insults by MAM and THC led to behavioral impairments observed in this study, which are consistent with several neuropsychiatric disorders including SCZ, they were not characterized by the same regional CBF alterations. The MAM model manifested increased CBF in the circle of Willis and the sensorimotor cortex together with decreased CBF in the hippocampus. On the other hand, the THC model showed no CBF changes. The source of the CBF changes remains unrevealed. It can be hypothesized that CBF could be preferentially dopamine regulated as already shown in brain cortex microcirculation^63,64^. Particularly, stimulation of D1 and D2 receptors induces vasodilation^63^, which may be reflected by increased CBF. Given that dopaminergic neural terminals occur regionally, we may suppose that also the CBF changes occur region-dependently. Therefore, the lack of CBF impairment observed in the THC model may also be due to selection of specific ROIs. Different models of neurodevelopmental pathology may feature specific CBF patterns in the brain. In this study, we assessed the same ROIs in both models but we cannot rule out the possibility of CBF alteration in different ROIs. Taken together, our data suggest that every neurodevelopmental model is characteristic by a specific pattern of regional CBF dysregulation with cortical regions being the most commonly altered as shown in the MAM and polyI:C models^18^. Future studies should reveal the causes of the ASL-detected perfusion changes, more specifically and confirm or reject our hypothesis of the major role of dopamine in the regulation of brain perfusion.
